# The Downside of Right Ventricular Apical Pacing

**DOI:** 10.1016/s0972-6292(16)30502-2

**Published:** 2012-05-20

**Authors:** Andrew Brenyo, Ilan Goldenberg, Alon Barsheshet

**Affiliations:** Division of Cardiology, Department of Medicine, University of Rochester Medical Center, Rochester, NY

**Keywords:** right ventricular pacing, down side

## Abstract

The right ventricular (RV) apex has been the standard pacing site since the development of implantable pacemaker technology. Although RV pacing was initially only utilized for the treatment of severe bradyarrhythmias usually due to complete heart block, today the indications for and implantation of RV pacing devices is dramatically larger. Recently, the adverse effects of chronic RV apical pacing have been described including an increased risk of heart failure and death. This review details the detrimental effects of RV apical pacing and their shared hemodynamic pathophysiology. In particular, the role of RV apical pacing induced ventricular dyssynchrony is highlighted with a specific focus on differential outcome based upon QRS morphology at implant.

## Introduction

The development of implantable pacemaker technology in the mid 20th century proved life saving for many patients with bradyarrhythmias [1]. In the intervening decades the indications for and volumes of permanent pacemaker placement have expanded [2-4] concurrent with a geographically dependent aging population. Right ventricular (RV) apical lead placement rapidly became standard practice secondary to the ease of site accessibility and lead stability. In most situations, RV apical pacing is effective and well tolerated.

However, an increasing amount of data has recently raised questions about the safety of RV apical pacing [5-14]. These safety concerns arise from the suggestion that RV apical pacing may have detrimental effects on cardiac structure and left ventricular (LV) function [15-17]. This is likely the result of the pathologic abnormal electrical and directly related abnormal mechanical activation of the ventricles seen as a consequence of RV apical pacing. An understanding of this pathophysiology has driven the development of pacing technology to limit the need for right ventricular apical pacing and the search for improved methods of ventricular pacing.

This review details the detrimental effects of RV apical pacing and its associated hemodynamic pathophysiology. In particular, the role of RV apical pacing induced ventricular dyssynchrony is highlighted with specific focus on differential outcome based upon QRS morphology at implant. Alternative and developing pacing strategies for patients with a permanent pacing indication including cardiac resynchronization therapy (CRT), alternative pacing sites, leads, programming configurations and energy sources are also discussed.

## RV Apical Pacing and Outcome: Clinical Data

For decades, RV apical pacing proved to be an effective therapy for sinus node disease [11], atrio-ventricular (AV) block [10], drug-refractory atrial fibrillation [18] and some forms of LV dysfunction [19]. A number of randomized studies have focused on the optimal pacing configuration and mode dependent on the pacing indication [6,10-14]. In addition to answering their intended questions, these studies have also shed light, although often indirectly, on the association between RV apical pacing and adverse cardiovascular outcome.

### Deleterious effects of RV apical pacing: Pacemaker Studies

Studies that have shown the deleterious effects of RV apical pacing can be categorized by the indication of pacemaker implantation and by the mode of pacing. Studies enrolling patients with sinus node dysfunction compared VVI with AAI pacing or DDD with AAI pacing whereas those enrolling patients with AV block compared the effects of DDD with VVI pacing.

Andersen et al [5] investigated 225 patients with sinus node dysfunction (SND), through a comparison of VVI to AAI pacing and found significantly higher cardiovascular mortality, incidence of heart failure (HF) and NYHA functional class in the ventricular pacemaker group.

Several clinical studies [14,20-22] in SND patients displayed that DDD pacing compared to AAI pacing induces left atrial dilation and, in the case of a high proportion of RV pacing, also reduces LV function, myocardial relaxation and myocardial blood flow. The DANPACE (Danish Multicenter Randomized Study on AAI Versus DDD Pacing in SND) study [13,14] was designed to prospectively determine if atrial pacing is superior to dual chamber pacing. DANPACE randomized 177 patients with isolated SND (without any significant atrio-ventricular conduction disturbance) to one of three pacing modes: AAIR, DDDR with a short atrio-ventricular (AV) delay (110-150 ms) and DDDR with a long atrio-ventricular delay (>250 ms). As expected, there was a significant difference in the frequency of ventricular pacing between modes: AAIR (0%), DDDR long AV (17%) and DDDR-short AV (90%). This programming dependent increase in frequency of RV pacing was associated with an unadjusted increased risk of atrial fibrillation (AF) and stroke. The risk for AF was lowest with atrial pacing: AAIR (3% per year), followed by DDDR-long (8.2% per year) and DDDR-short (11.7% per year), and there was a trend for fewer thrombo-embolic events with atrial pacing: AAIR (1.9% per year), followed by DDDR-long (2.2% per year) and DDDR-short (4.0% per year) [14].

Providing a possible mechanism for the increase in both endpoints, Nielsen et al. demonstrated that the use of dual-chamber pacing (i.e. high RV pacing) is associated with left atrial enlargement when compared to atrial pacing alone [14]. This suggests that one of the deleterious effects of right ventricular pacing may be increased atrial pressure, resulting in structural atrial remodeling and increased risk of AF.

Several studies of DDD vs. VVI only pacing in patients with AV block [10,11], or both AV block and SND [12], generated the hypothesis that RV apical pacing may be detrimental (Table 1). The expectation was that DDD pacing would be beneficial over RV apical only (VVI) pacing secondary to maintenance of AV synchrony, resulting in a reduction in heart failure (HF), cardiovascular mortality, AF and stroke. Within these studies, only AF was significantly reduced with DDD pacing leaving questions regarding the importance of maintaining AV synchrony on heart failure and mortality.

A subsequent subgroup analysis of MOST (MOde Selection Trial) among patients with QRS < 120 msec shed light on a possible underlying reason for the negative outcomes of these studies [11]. It demonstrated a strong association between RV pacing and the risk of HF events and AF in both DDD (mean 90% RV pacing burden, n = 707) and VVI pacing (mean 50% RV pacing burden, n = 632) groups. More specifically, patients with greater than 40% of ventricular pacing burden in the DDD group and > 80% of ventricular pacing in the VVI pacing group had more than two fold increased risk for HF events (DDD adjusted hazard ratio (HR): 2.60; 95% confidence interval (CI): 1.05 to 6.47; p < 0.05; VVI HR: 2.50; 95% CI: 1.44 to 4.36; p < 0.05). Similarly, each 25% increase in RV pacing burden was associated with an approximate mean increase of 28% (adjusted 36% for DDD and 21% for VVI only) in AF. The similar increase in HF and AF with DDD and VVI only pacing supports the notion that maintenance of AV synchrony does not convey a risk reduction in either. Rather, the RV apical pacing burden outweighed any benefit of AV synchrony in the DDD group and was the primary driver behind the negative trial result. Freudenberger et al, [23] examining more than 11,000 patients who underwent pacemaker implantation, found that permanent dual chamber or ventricular pacing in patients who did not have HF before implantation, significantly increased their risk for HF hospitalizations or HF-related deaths compared with matched control group.

### Deleterious effects of RV apical pacing: Defibrillator Studies

The DAVID (Dual Chamber and Implantable Defibrillator) trial further substantiated the association between RV pacing and adverse cardiovascular outcome [6]. Operating with the hypothesis that DDD pacing will convey a reduction in HF, 506 patients with a standard indication for ICD implant and no indication for pacing were randomized to "physiologic pacing" ICD with DDD backup heart rate 70 (DDD -70) vs. single chamber ICD with VVIR backup heart rate 40 (VVI - 40). After one year follow up, the combined endpoint of hospitalization for HF or death was significantly higher for the DDD - 70 group (26.7%) compared to the VVI - 40 group (16.1%) with an adjusted HR of 1.61 (95% CI 1.06-2.44, p = 0.03). The difference in the backup rate between the two groups resulted in a marked difference in the burden of RV pacing with DDD - 70 patients paced 60% of the time and VVI - 40 patients just 3% of the time. Congruent with findings from MOST, an RV pacing dose dependent positive relationship with adverse cardiovascular events was noted.

The MADIT II (Multicenter Automated Defibrillator Implantation Trial II) enrolled 1,232 patients with ischemic cardiomyopathy randomized to ICD vs. medical therapy in a 3:2 ratio. ICD configurations within the study included single chamber programmed VVI-40 (44%) and dual chamber programmed DDD-70 (56%). Steinberg et al reported the short term (median 1.5 years) follow up of RV pacing in the ICD arm dichotomized by a pacing burden of greater (high RV pacing) and less than 50% (low RV pacing) [8]. Patients with high RV pacing were older, had higher blood urea nitrogen levels, and were more likely to have wide QRS and LBBB compared with non-ICD patients or patients with low RV pacing. After multivariate adjustment, high RV pacing patients were at significantly increased risk of new or worsened HF (HR 1.93, p = 0.002) and appropriate ICD therapy for VT/VF (HR 1.50, p = 0.02). However, the mortality rates were similar for high (13%) and low (10%) RV pacing groups (adjusted HR 1.07, p = 0.78) [8].

**Figure 1:**
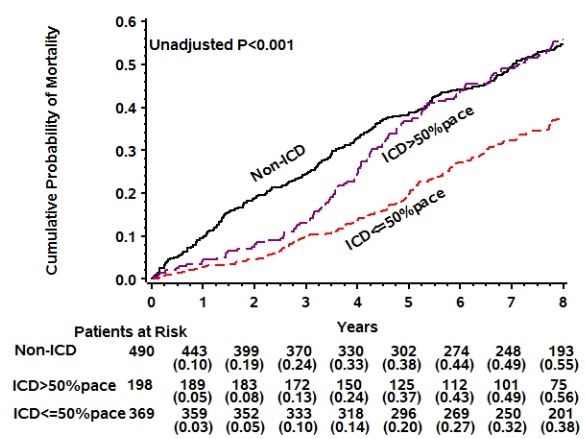
Probability of long-term mortality by percent of right ventricular pacing and ICD implantation in the MADIT II trial with extended follow up. (reproduced with permission of the publisher [9])

Most recently, we have analyzed the association between percent RV pacing and long term mortality in MADIT II during an 8-year follow-up [9]. Patients were categorized into three subgroups: low RV pacing (< 50% pacing, n = 369), high RV pacing (≥ 50% pacing, n = 198), and no ICD (n = 490). During the first 3 years after enrollment, the benefit of the ICD was prominent both in patients with low RV pacing (adjusted 65% reduction in risk of death, P<0.001) and in those with high RV pacing (adjusted 62% reduction in risk of death, P<0.001); In contrast, during the late phase of the extended follow-up period (4-8 years) ICD therapy was associated with a significant survival benefit among patients in the low RV pacing subgroup (adjusted 40% reduction in mortality risk, P = 0.001) but not in the high RV pacing subgroup (adjusted HR = 0.89, P = 0.45). In addition, during the total 8 year follow up, high RV pacing was shown to be associated with a significant adjusted 40% (P = 0.01) increase in the risk of death compared with low RV pacing (Figure 1). Thus, the long-term benefit of an ICD in reducing mortality is prominent in patients with low RV pacing but attenuated in patients with high RV pacing, and patients with high versus low RV pacing have increased long term mortality risk. A reasonable interpretation of these findings is that frequent RV pacing resulted in ventricular dyssynchrony, development and deterioration of HF which takes several years to be translated into increased mortality.

## The Pathophysiology of RV Apical Pacing

The deleterious effects of RV apical pacing have been attributed to the abnormal electrical and mechanical activation induced secondary to this form of pacing. During RV apical pacing, the electrical wave front propagates through the myocardium, rather than through the His-Purkinje conduction system. As a result, the electrical wave front propagates more slowly and induces heterogeneity in electrical activation of the myocardium, comparable but not identical to left bundle branch block. This is characterized by wave front breakthrough at the interventricular septum and latest activation at the infero-posterior base of the left ventricle [24-26].

Similar to the changes in electrical activation of the ventricles, the mechanical activation pattern is altered during RV apical pacing. Importantly, not only does the anatomic onset of mechanical contraction differ, but also the resulting pattern of mechanical contraction [27]. Badke et al [28] detailed how apical pacing is associated with a diminished rate of change in left ventricular pressure (dP/dt) and an abnormal dyssynchronous contraction pattern. The paced region contract early at a time of low load, but then is stretched later in systole as the lateral wall finally contracts [28-29]. Hemodynamically, asynchronous myocardial contraction significantly decreases the stroke volume and right-shifts the left ventricular end-systolic pressure - volume relationship. Mismatch between the relaxation of early- and late-contracting regions leads to a decrease in left ventricular filling time. Thus, RV apical pacing leads to ventricular dyssynchronization, systolic and diastolic ventricular dysfunction, increase in wall stress and energetic inefficiency [29].

Beyond the hemodynamic effects of ventricular dyssynchrony, it has become clear that long-term RV pacing may also result in structural changes and adverse LV remodeling. Originally reported in1986 in an RV pacing dog complete heart block model [30], three months of RV pacing resulted in myofibrillar disarray in 75% of these dogs. In cardiomyopathies induced by high-rate right ventricular apical pacing, they observed significant differences in the expression of proteins involved in myocyte contraction, which were not seen in high-rate atrial-pacing-induced cardiomyopathies with preserved ventricular synchrony [31]. The lateral left ventricular free wall (late-activated) displayed the most pronounced cellular derangements, such as down-regulation of protein kinases, proteins involved in calcium homeostasis and intercellular connections [31]. In addition, changes in LV wall thickness (the early activated wall becomes thinner whereas the late activated wall becomes thicker) [17], LV remodeling [32], left atrial remodeling [33], functional mitral regurgitation [34], and perfusion abnormalities [35-36] all appear to play a role in the pathophysiology of RV apical pacing; predominately as downstream consequences of iatrogenic ventricular dyssynchrony.

## RV Pacing and QRS morphology at implant

It has long been known that native LBBB can have profound hemodynamic effects due to ventricular dyssynchrony, particularly among patients with HF [37]. As detailed above, ventricular pacing contributes to the development or exacerbation of HF by producing an iatrogenic form of LBBB and ventricular dyssynchrony reducing systolic and diastolic ventricular function. Mechanical activation in patients with chronic RV pacing has been compared to those with native LBBB, with patients from both groups having intraventricular dyssynchrony, but RV pacing patients displaying greater interventricular dyssynchrony and more often had sites of earliest activation from the apex and inferior septum [38].

**Figure 2:**
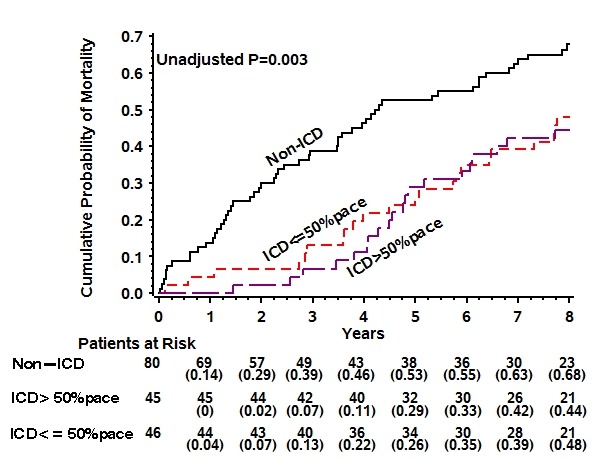

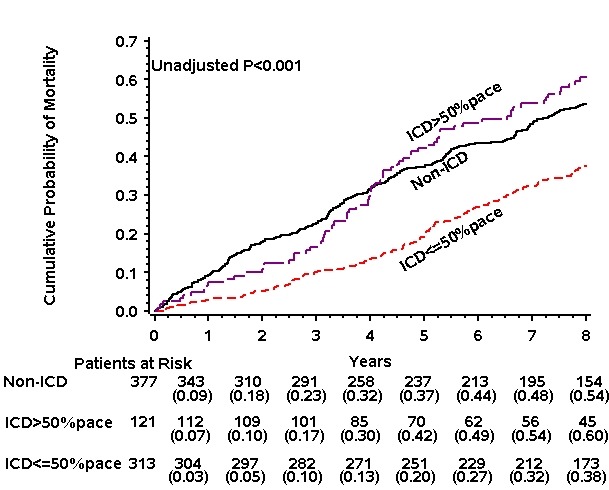
Probability of long-term mortality by percent of right ventricular pacing in patients with LBBB (A) and without LBBB (B) in the MADIT II trial with extended follow up. (reproduced with permission of the publisher [9])

We explored the effects of RV pacing on ICD benefit according to LBBB at enrollment in MADIT-II during an extended long term follow-up of 8 years [9]. We found that high RV pacing was associated with increased long term mortality only in patients who did not have LBBB at baseline (Figure 2). Among patients without LBBB, high RV pacing was associated with an adjusted 63% (P = 0.002) increase in mortality risk compared with the low RV pacing subgroup, whereas among patients with LBBB there was no significant difference in mortality during long-term follow-up between high and low RV pacing patients (High vs. low RV pacing adjusted HR = 0.74, p=0.343; P value for interaction [QRS morphology by RV pacing burden] = 0.024). Consistent with our findings, Saad et al [39] showed in a small study (including 44 HF patients, 12 with LBBB) that high RV pacing is associated with poor outcome only in the absence of LBBB. These findings may suggest that dyssynchrony induced by RV apical pacing is somewhat worse than naturally occurring LBBB dyssynchrony. Alternatively, these findings may only suggest that RV pacing may not be harmful to patients with systolic HF and LBBB, as the incremental dyssynchrony induced by RV pacing is less significant.

In contrast to these findings, Hayes et al [40] in a substudy of the DAVID trial, found that patients programmed to high pacing volume (DDD-70) with an abnormal QRS duration (≥ 110 msec) had an unadjusted 25% increased risk of death or hospitalization for HF (p = 0.01). Breaking down the abnormal QRS duration group into BBB morphologies, the adverse outcome for the group as a whole appeared to be driven by the LBBB patients (30%) that experienced an unadjusted 40% increase in HF or death (p = 0.03).

Reconciling the stark differences among these studies remains difficult. The primary differences between the MADIT-II substudy and the DAVID substudy include different study endpoints (death in MADIT II vs. HF or death in DAVID), duration of follow up and indication for pacing. DAVID specifically randomized patients based upon pacing programming where the focus of MADIT II was the efficacy of primary prevention ICD therapy in ischemic cardiomyopathy. Within the MADIT II sub-analysis the primary limitation was the clinical differences between high and low RV pacing subgroups. We cannot completely exclude the possibility that sicker patients required more pacing and had poorer outcomes as a result. Furthermore, data on percent RV pacing were collected only among 79% of the 720 patients who received an ICD in MADIT II. Patients who were not included in this data analysis due to missing pacing information appeared to be sicker with an elevated in-trial mortality rate of 42% compared with 12% in patients analyzed in our study. The DAVID analysis was limited by size and follow up duration.

## Minimizing the Detrimental Effects of RV Pacing

Methods to avoid the detrimental effects of RV pacing include device programming to minimize RV pacing, alternative pacing sites, and biventricular pacing. The current mainstay of programming based methods to avoid RV pacing includes AV search or MVP (managed ventricular pacing) mode. Such programming was evaluated in the SAVE PACe (Search AV Extension and Managed Ventricular Pacing for Promoting Atrioventricular Conduction) trial, where 1,065 patients with sinus node disease and intact AV conduction were randomized between conventional dual-chamber pacing and dual-chamber minimal ventricular pacing [41]. RV pacing burden was significantly reduced with MVP, compared with conventional dual-chamber ventricular pacing (9.1% vs. 99.0%, p < 0.001). After a mean follow-up of 1.7 years, the unadjusted development of persistent atrial fibrillation was significantly reduced with MVP (7.9% in minimal RV pacing vs. 12.7% in conventional dual-chamber pacing, p = 0.004). No significant difference in HF or mortality was noted with MVP programming.

When RV pacing is inevitable, the His bundle [42,43], right ventricular outflow tract [44,45] and right ventricular septum [46,47] have been studied as alternate pacing sites. Selectively pacing these sites may reduce pacing induced ventricular dyssynchrony [48-50] and potentially result in a reduction in ventricular volumes with improved LV function compared to RV apical pacing. However, with the small size, short term follow up and the absence of a reduction in HF or death within the randomized prospective studies, the benefit of alternative site RV pacing remains controversial.

For RV apical pacing dependent patients with abnormal LV function [51-56] or RV apical pacing associated HF [57,58], biventricular pacing has emerged as a viable option to minimize the detrimental effects of RV pacing. Although some studies have demonstrated a clear long-term benefit of CRT over RV pacing with regard to peak VO2 or functional class [51,53], others have demonstrated only modest [54,55,57,58] or minimal benefit [56].

The PACE trial [59] directly examined the efficacy of biventricular pacing within this population. Patients post biventricular pacemakers (177) were prospectively enrolled and randomized to receive biventricular pacing (89 patients) or RV apical pacing (88 patients). No significant differences in LV ejection fraction (61.5 ± 6.6% for RV pacing, 61.9 ± 6.7 for biventricular pacing, p = 0.86) or LV end systolic volume (28.6 ± 10.7ml for RV pacing, 28.6 ± 9.4ml for biventricular pacing, p = 0.71) were present at baseline. At 12 months, the mean LV ejection fraction was significantly lower in the RV pacing group than in the biventricular-pacing group (54.8 ± 9.1% vs. 62.2 ± 7.0%, p < 0.001). Similarly, the LV end-systolic volume was significantly higher in the RV pacing group than in the biventricular-pacing group (35.7 ± 16.3 ml vs. 27.6 ± 10.4 ml, p < 0.001), with a relative change from baseline of 25% (p < 0.001). These results support the detrimental effect of RV apical pacing manifest through adverse LV remodeling and deterioration in LV function with such effects prevented by biventricular pacing.

## Conclusions

Right ventricular apical pacing is an integral part of the treatment of brady-arrhythmias for the majority of patients receiving pacemakers. Right ventricular apical pacing is, however, an often pathologic substitute for intrinsic ventricular activation over the His-Purkinje system. Several reports indicate that this form of pacing is detrimental potentially increasing the risk of heart failure episodes and death, particularly in patients with abnormal LV function. Further studies are needed to clarify the mechanisms underlying the deleterious effects of RV pacing, RV apical pacing burden to be avoided, and the specific risk factors for poor outcome among patients with high RV pacing burden. In the meantime, alternative pacemaker programming and configurations are available that can minimize the frequency and detrimental nature of ventricular pacing in many pacemaker patients. Additional research will determine if different forms of ventricular pacing, such as right ventricular outflow tract, RV septal pacing, or biventricular pacing will improve outcomes in patients who require ventricular stimulation.

## Figures and Tables

**Table 1:**
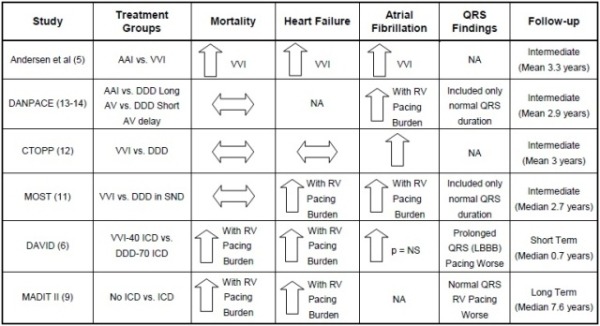
Pacing and ICD studies examining RV pacing and Outcome
